# The Processing of Causal and Hierarchical Relations in Semantic Memory as Revealed by N400 and Frontal Negativity

**DOI:** 10.1371/journal.pone.0132679

**Published:** 2015-07-06

**Authors:** Xiuling Liang, Qingfei Chen, Yi Lei, Hong Li

**Affiliations:** 1 Research Centre for Brain Function and Psychological Science, Shenzhen University, Shenzhen, China; 2 Research Centre for Special Economic Zone Research, Shenzhen University, Shenzhen, China; Max Planck Institute for Human Cognitive and Brain Sciences, GERMANY

## Abstract

Most current studies investigating semantic memory have focused on associative (ring-emerald) or taxonomic relations (bird-sparrow). Little is known about the question of how causal relations (virus- epidemic) are stored and accessed in semantic memory. The goal of this study was to examine the processing of causally related, general associatively related and hierarchically related word pairs when participants were required to evaluate whether pairs of words were related in any way. The ERP data showed that the N400 amplitude (200-500ms) elicited by unrelated related words was more negative than all related words. Furthermore, the late frontal distributed negativity (500-700ms) elicited by causally related words was smaller than hierarchically related words, but not for general associated words. These results suggested the processing of causal relations and hierarchical relations in semantic memory recruited different degrees of cognitive resources, especially for role binding.

## Introduction

The ability to use knowledge of causal relations to plan, act and reason is one of the most fundamental attributes of human cognition [1]. Neural studies have begun to use a variety of neuroimaging techniques to investigate brain areas that appear sensitive to causal processing [2], such as causal perception [3,4], learning new causal relations [5,6] and causal inferences in text processing [7]. Generally speaking, some types of causal relations present in the environment may be immediately apparent through perceptual causation (e.g., causal perception), whereas other types of causal relations are likely learned through experience [2]. The present study sets out to further investigate how stored causal relations (along with non-causal associations) are represented and accessed in semantic memory.

Most previous studies about semantic relations have mainly focused on the processing of taxonomic relations within the same or different hierarchical levels, such as bird—robin [8,9], or thematic relations which includes externally or complementary related items within scenarios or events [10–12]. For example, Chen et al (2014) investigate the automatic processing of hierarchical relation (e.g., offspring—grandson, offspring include grandson) and productive relation (e.g., bee—honey, bees produce honey) in semantic priming via ERPs. The results found that the frontal negativity elicited by productive relations was smaller than hierarchical relations. They suggested that productive relations played an increased salience in knowledge structure relative to less prominent hierarchical relations.

However, the causal relation, as well as productive relation, was only regarded as examples of thematic relations by earlier research [13], and there are a few studies on the dissociation of causal relations and taxonomic relations. Until recently, several studies have explored how causal relations are represented and accessed in semantic memory [14–16]. For example, participants were faster to decide whether two words were causally related when the first word referred to a cause and the second word referred to its effect (e.g., *spark* prior to *fire*) than vice versa (*fire* prior to *spark*) [14]. However, such reaction time (RT) advantage was not found when participants were required to verify whether same word pairs were general associatively related. Recently, researchers found that causal relationships were verified faster if “cause” appeared vertically above “effect” than the reverse, as well as when cause horizontally preceded effect than the reverse [15]. However, the hierarchical relationships were verified faster only when the “superordinate-level” appeared vertically above “subordinate-level” than the reverse. They suggested that the causal asymmetry was mainly based on temporal order, whereas the representation of hierarchical asymmetry was based on spatial arrangement.

Additional studies have adapted the paradigm introduced by Fenker et al. (2005) to investigate the neural basis of causal relationship processing through fMRI [17,18]. For example, when participants were required to determine whether pairs of words were causally related (e.g., virus—epidemic) or associatively related (e.g., emerald—ring) in different blocks of trials, or in same blocks of trials, the results suggested that evaluating causally related words generated distinct activation in left dorsolateral prefrontal cortex and right precuneus known to code role binding [18], as well as OFC, amygdala, striatum, and substantia nigra/ventral tegmental area known to code prediction errors [17], beyond those used during associatively related words, suggesting that role binding or prediction processing might participate the processing of causal relations. Overall, these results suggested that the processing of causal relations was dissociated from the processing of more general associative relations. Evaluating a causal relation may require the formation of a working-memory representation, in which the specific roles of cause and effect are distinguished. For example, a virus can cause an epidemic, whereas an epidemic does not cause a virus. Thus verifying a causal relation will not only depend on simple associative priming between two words in semantic memory, but also require binding the relevant events into “cause” and “effect” roles. However, little is known about the time course of how stored causal relations are represented and accessed in semantic memory.

Additionally, previous studies that have investigated causally related words tended to compare the relationship with non-causal associative relationships. Although this comparison has yielded valuable insights, the associative relations used for comparison in previous studies have often been diverse; being composed of a variety of relationships, including taxonomic and thematic relations. This may have affected the results because the processing of thematic and taxonomic relations has also been shown to dissociate in adults [11,19,20]. Furthermore, as mentioned earlier, the causal relations was also regarded as an example of thematic relations [13]. Therefore, not using pure semantic related word pairs as a control makes any inferences regarding differentiation between these types of relations beyond the scope of their investigation. Based on our previous studies [11,15], we added a hierarchical relation as a controlled condition, in which the pairs included objects represented by a basic-level concept and objects represented by a subordinate concept in a category (e.g., birds include sparrow, robin, etc. [21].

In the present study, causal relations, hierarchical relations, and general associative relations were compared to tease apart potential differences in representations of the relationships using event related potentials (ERPs). The use of ERP data yields a more direct measure of the processing stages that are affected by stimulus manipulations [22]. ERP data is here considered as just complementary to behavioral and fMRI studies in furthering our understanding of these differences. The present study considered a well investigated ERP component, N400, as a potentially good physiological index for exploring this issue.

The N400 component has been shown to be sensitive to the strength of semantic relations [23,24]. For example, a larger N400 was elicited by semantically unrelated prime-target word pairs than semantically related word pairs [25,26]. Furthermore, the N400 is also highly sensitive to different types of semantic relationship, such as thematic vs. causal relations, independently of the strength of semantic associations [27–29]. For example, researchers found that the N400 was smallest for directly causal relations, greater for indirectly causal relations, and biggest for non-causal relations, while keeping semantic association constant across conditions [27]. That is, a larger N400 was observed in the case in which there was not clear causal relationship between the two sentences (As in, 'Jill tanned well and put on sunscreen. She had sunburn on Monday.')

Based on the above analysis, the main goal of this study is to examine how stored causal relations are represented and accessed in semantic memory, and the dissociation between causal, hierarchical and associative relations using ERPs. To elicit implicit processing and to minimize strategic influences, we used general relationship judgment task, in which no RT advantage was found for the processing of causal relations [14]. Specifically, participants were presented with causally related, associatively related, hierarchically related and unrelated word pairs, and required to evaluate whether the two words were related in any way as quickly as possible. If the processing of related words differs from unrelated words, the N400 amplitude elicited by unrelated words should be consistently larger than related words. Furthermore, if the causal relations was as an example of thematic relations [13], similar difference between the processing of hierarchical relations and productive relations, such as frontal negativity, should be found between the processing of causal relations and hierarchical relations [11].

## Methods

### Participants

Sixteen healthy right-handed participants (8 female), ranging in age from 19 to 22 years, took part in the main experiment who had not previously participated in the rating study. The study was approved by the University’s ethics committee. Data from one participant were discarded due to excessive EEG artifacts.

### Stimuli

The stimuli consisted of 160 pairs of 4 character Chinese words. Before the main experiment, 200 items were initially normed. That is, 50 causally related (e.g., virus—epidemic), 50 hierarchically related (e.g., fish—goldfish), 50 non-causal associatively related (e.g., ring—emerald) and 50 unrelated (e.g., door—pinball) word pairs were selected from previous studies and translated into Chinese [14,21]. To increase the ratio of unrelated pairs, in order to balance required responses, the unrelated filler word pairs was created in which the word pairs in related conditions were re-paired to form another sub-list of 150 unrelated pairs (50 word pairs for each condition; e.g., fish—pine, [30]. Subsequently, fifty-nine healthy undergraduate students (not included in the main study) were recruited and paid to participate in several normative studies to account for the associative strength and the strength of statistical contingency, which might affect the comparison between causal judgments and hierarchical judgments.

At first, 13 subjects evaluated the words in a preliminary phase, in which they marked any words that they had not heard before. Words that were marked by two or more participants were replaced. One word pair (protestants-baptist) was replaced with new pair (temple-monk) because of the cultural difference.

After the initial rating of materials, 23 undergraduates participated in a specific strength test. In the causal strength test, participants were required to rate the likelihood that the event or object described by the first word caused the event or object described by the second word on a 7 point scale, where 7 indicated the highest likelihood. In the hierarchical strength test, participants were required to rate the degree to which the event or object described by the first word included the event or object described by the second word. In the general associative relationship test, participants were required to rate the strength of the meaningful relationship between the two items. The unrelated word pairs were also rated on the strength of general associative relationship. For example, the word pair “virus—epidemic”, received a typical rating of “6” or “7” on the causally relatedness scale; while the word pair “fish—goldfish” received a typical rating of “6” or “7” on the hierarchically relatedness scale. Furthermore, the word pair “ring—emerald”, received a typical rating of “5” or “6” on the associatively relatedness scale, while the word pair “door—pinball” received a typical rating of “1” or “2” on the associatively relatedness scales.

As a next step, another 23 undergraduates participated in a general associative strength test for the above causally related, hierarchically related, and unrelated words, just like the earlier general associative relationship test for associatively related words. Furthermore, we conducted a norming task to account for the strength of statistical contingency between our items, as these measures sometimes affect the associative strength between items [14]. 23 participants were presented with the above 200 word pairs on a computer screen using E-prime software. All types of related and unrelated word pairs were presented, and for each pair participants were required to estimate that if the object or event described the first of the two words occurred 100 times, how many times the object or event described by the second word would occur. For example, “if *bird* occurs 100 times, how often does *sparrow* occur?” Participants were required to rate co-occurrence on a scale from 0 to 100, in increments of 10.

Based on the above ratings, 40 from each of the causally related, hierarchically related, associatively related words with high strength and statistical frequency, as well as 40 unrelated pairings with low strength and statistical frequency, were selected as stimuli for the Experimental task (Table 1). There was a significant main effect of type of relation (causal relation, hierarchical relation, associative relation and unrelated words) on the specific strength test, *F* (3, 88) = 151.94, *p* < .001, *η* ^*2*^ = .84. Similarly, we also compared the general associative strength test for causally related, hierarchically related, associatively related, and unrelated words. There was a significant main effect of type of relation on the general associative strength test, *F* (3, 88) = 148.17, *p* < .001, *η* ^*2*^ = .81. Post hoc pair-wise comparison indicated that the associative strengths of unrelated word pairs were significantly lower than other three related conditions in both specific and general strength tests (*ps* < .001), whereas there was no significant difference among causally related, associatively related, and hierarchically related word pairs, *ps*>.90.

**Table 1:** The mean strengths and statistical frequency and standard deviations over subjects and stimuli.

	Specific strength test		General associative strength		Statistical frequency ratings	
	*M*	*SD*	*M*	*SD*	*M*	*SD*
Hierarchically related	5.58	1.01	5.80	.98	56.60	10.47
Causally related	5.46	0.76	5.56	.75	58.15	12.32
Associatively related	5.49	0.88	5.49	0.88	55.82	8.58
Semantically unrelated	1.51	0.25	1.61	.46	21.27	15.72

Furthermore, the results found a significant main effect of type of relation on the statistical frequency ratings, *F* (3, 88) = 50.18, *p* < .001, *η* ^*2*^ = .63. Post hoc pair-wise comparison indicated that the statistical frequency of unrelated word pairs was significantly lower than other three related conditions (*ps* < .001), whereas there was no significant difference among causally related, associatively related, and hierarchically related word pairs, *ps*>.90.

The mean word frequencies, based on a current Chinese language database (Center for Chinese Linguistics PKU, China), did not differ significantly among the four groups of words, *F* (3, 316) = .53, *p* = .67, *η* ^*2*^ = .005. They were 3515 (*SD* = 5095) for causally related words, 3998 (*SD* = 5797) for noncausally associatively related words, 3068 (*SD* = 3918) for hierarchically related word pairs, and 3888 (*SD* = 5739) for unrelated words.

### Procedure

All related (causal, non-causal associative, hierarchical) word pairs were repeated twice, the unrelated word pairs were repeated three times and the unrelated filler word pairs were presented only once, and participants were required to evaluate whether the presented word pairs were related in any way. Thus, all the words used in related and unrelated conditions were presented three times, and four hundred and eighty word pair trials were used in this study. These trials were distributed as follows: 80 causally related word pairs, 80 associatively related word pairs, 80 hierarchically related word pairs, 120 unrelated word pairs, and 120 unrelated filler word pairs.

**Fig 1:**
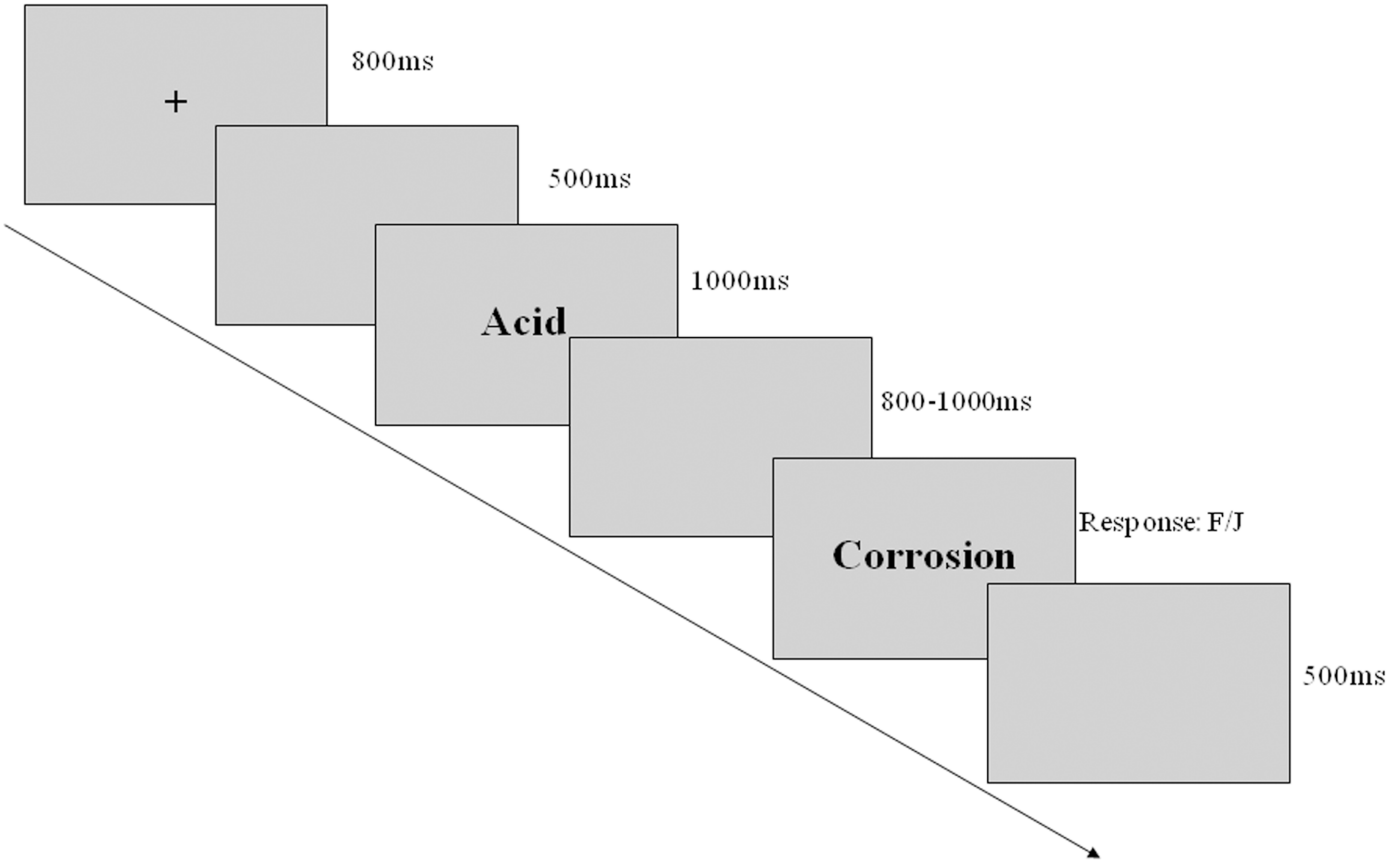
Illustration of the experimental procedure (causally related condition).

The words were presented sequentially on a computer screen using E-prime software. As showed in Fig 1, a fixation (“+”) was presented in the center of a gray screen for 800 ms at the beginning of each trial. Subsequently, the first word was presented for 1000 ms, followed by a blank screen with random duration (800–1000 ms). Next, the second word appeared on the screen and remained until participants made a response. Subjects were instructed to respond rapidly and accurately to the second word, and make a “yes” or “no” response by pressing one of two keys (“F” or “J”) with the left or right index finger. The use of “F” and “J” for “yes” or “no” response was counterbalanced across subjects. Sixteen practice trails were used to familiarize subjects with the procedure. The practice trials were selected from the unused 40 word pairs that were not included in the primary experiment.

### ERP recordings and data analysis

Brain electrical activity was recorded from 64 tin electrodes mounted on an elastic cap, with a ground electrode on the medial frontal line and references on the left and right mastoid [22,31]. The vertical electro-oculograms (EOGs) were recorded from the left eye supra-orbitally and infra-orbitally. The horizontal EOG was recorded from the orbital rim of both eyes. The impedance of all electrodes was maintained below 10 kΩ. The signals were amplified by a 0.05–100 Hz band pass and continuously sampled at 500Hz/channel for offline analysis. All the bioelectric signals were analyzed off-line using Brain vision analyzer 2.0 (Brain Products, Germany). The signal was passed through a 0.1 to 35 Hz digital band-pass filter. Artifacts such as blinks and eye movements were eliminated offline using ocular correction ICA.

Both the EEGs elicited by the first word and the second word were time-locked. Data were segmented into epochs of 1000ms duration, including a 200ms baseline. Additionally, after the data were baseline corrected, trials with artifacts due to muscle potentials, or amplifier blocking were eliminated, as were trials with peak-to-peak deflections exceeding ± 80 μV. As a result, less than 7% of the data were lost due to artifacts, muscle potentials, and so on. However, no significant differences were found for the ERP waves elicited by the first word. Thus, only EEGs elicited by the second word were examined.

Based on the grand average waveforms and topographies, we focused our analysis on the N400 peaking between 300 ms and 500 ms, and the later ERP effect peaking between 500 ms and 700 ms. Similar to our previous study [11], repeated ANOVAs were conducted on mean amplitude of 200–500ms and 500–700ms with type of relation, laterality (three levels, left, middle and right sites) and frontality (five levels, frontal: left—F3, middle—Fz, right—F4; frontal central: left—FC3, middle—FCz, right—FC4; central: left—C3, middle—Cz, right—C4; central parietal: left—CP3, middle—CPz, right—CP4; parietal: left—P3, middle—Pz, right—P4) as repeated factors. Statistical tests were computed with the SPSS software package 19.0 (IBM, USA). The degrees of freedom of the *F* ratio were corrected for violations of the sphericity assumption according to Greenhouse Geisser for all analyses, and Bonferroni corrections were used for each comparison. The significant difference criterion was set to *p* = .05.

## Results

### Behavioral Results

**Fig 2:**
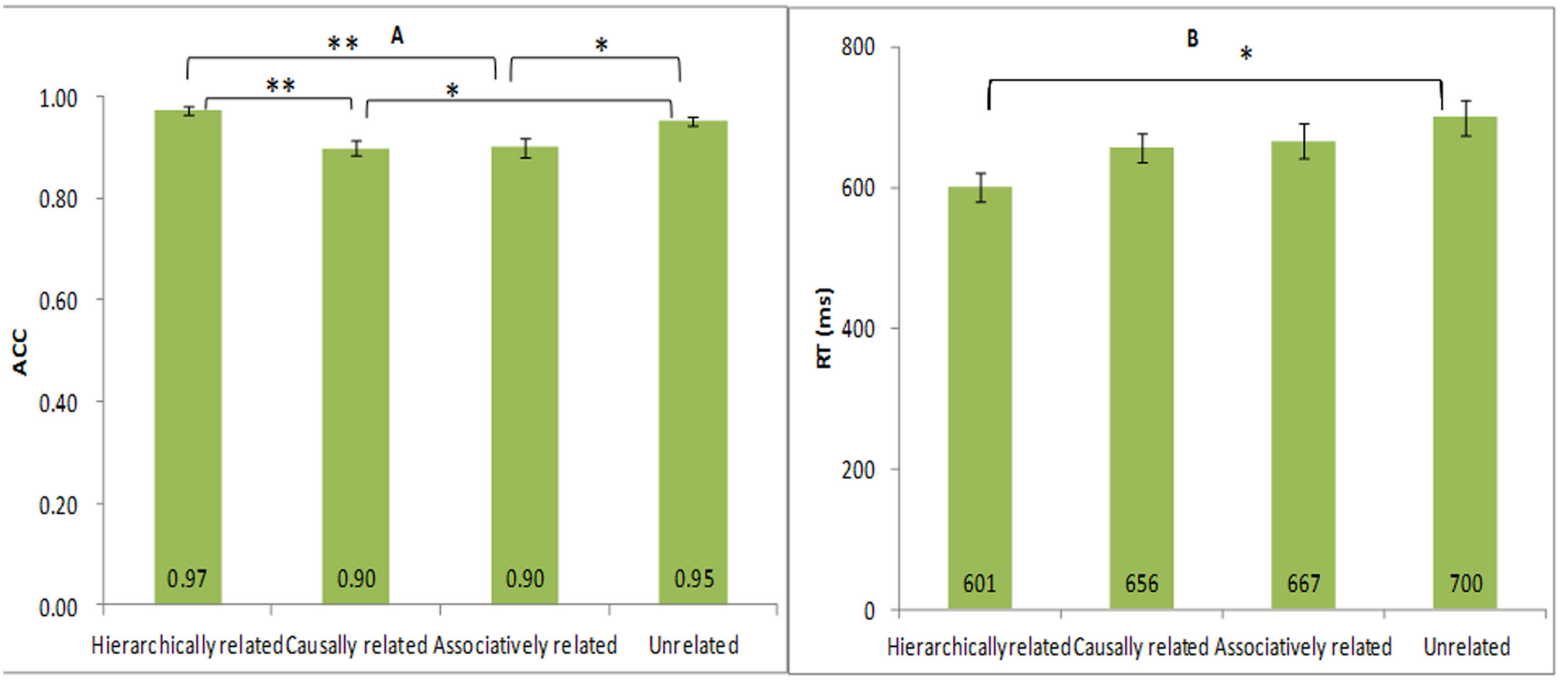
Mean accuracy rates (A, M±SE) and reaction times (B, M±SE) in the different conditions. The accuracy rates for hierarchically related and unrelated words were higher than causally related and associatively related words. However, the difference on RT was only found between hierarchically related and unrelated words.

Mean reaction times (RT) and accuracy rates (ACC) were presented in Fig 2 and were analyzed by separate one-way ANOVAs. There was a significant main effect of type of relation on the ACC, *F*(3, 56) = 7.71, *p <* .001. Post hoc pair-wise comparison showed that the ACC for hierarchically related (*M* = .97, *SD* = .04) were higher than causally related (*M* = .90, *SD* = .05) and associatively related (*M* = .90, *SD* = .08) words, *ps* < .002. The ACC for unrelated words (*M* = .93, *SD* = .06) was also higher than causally related and associatively related words, *ps* < .05. No other significant was found among other conditions, *ps*>.90.

Furthermore, the ANOVA revealed significant differences among different conditions on the RT, *F* (3, 56) = 3.32, *p* = 0.03. Post hoc pair-wise comparison showed that the RT for hierarchically related (*M* = 601ms, *SD* = 79ms) were shorter than unrelated words (*M* = 700 ms, *SD* = 90 ms), *p* = .02. Although the absolute data of RT for hierarchically related words (*M* = 601ms, *SD* = 79ms) were shorter than causally related (*M* = 656 ms, *SD* = 79 ms) and associatively related words (*M* = 667ms, *SD* = 94 ms), no significant differences were found among them, *ps* >.26. No other significant difference was found among other conditions, *ps*>.90.

### ERP Results

With regard to the repetition of items, Renoult (2010) have found that semantic processing and its neural bases are similar in repeated and non-repeated conditions. What is more, using repeated words in explicit semantic designs has the advantage of avoiding problems of category specificity and physical variance that are unavoidable when using large groups of words. To better address this issue, we have labeled the word pairs as different marks when they are presented for the first time and when they are repeated in separate blocks.

Before merging two types of stimulus together, we analyzed them and no significant difference was found between them. Specifically, for the N400 at 300–500ms, there was no significant difference for the repetition effect for casually related words: *F* (1, 14) = 1.27, *p* = .28, *η*_*p*_^*2*^ = .08, hierarchically related words: *F* (1, 14) = 2.10, *p* = .17, *η*_*p*_^*2*^ = .13, associatively related words: *F* (1, 14) = 1.56, *p* = .23, *η*_*p*_^*2*^ = .10, and unrelated word pairs: *F* (1, 14) = .24, *p* = .63, *η*_*p*_^*2*^ = .02. Similarly, for the frontal negativity at 500–700ms, there was no significant difference for the repetition effect for casually related words: *F* (1, 14) = 3.43, *p* = .09, *η*_*p*_^*2*^ = .19, hierarchically related words: *F* (1, 14) = .77, *p* = .40, *η*_*p*_^*2*^ = .05, associatively related words: *F* (1, 14) = .05, *p* = .83, *η*_*p*_^*2*^ = .004, and unrelated word pairs: *F* (1, 14) = .001, *p* = .98, *η*_*p*_^*2*^ = .0001.

### The N400 (200–500 ms)

**Fig 3:**
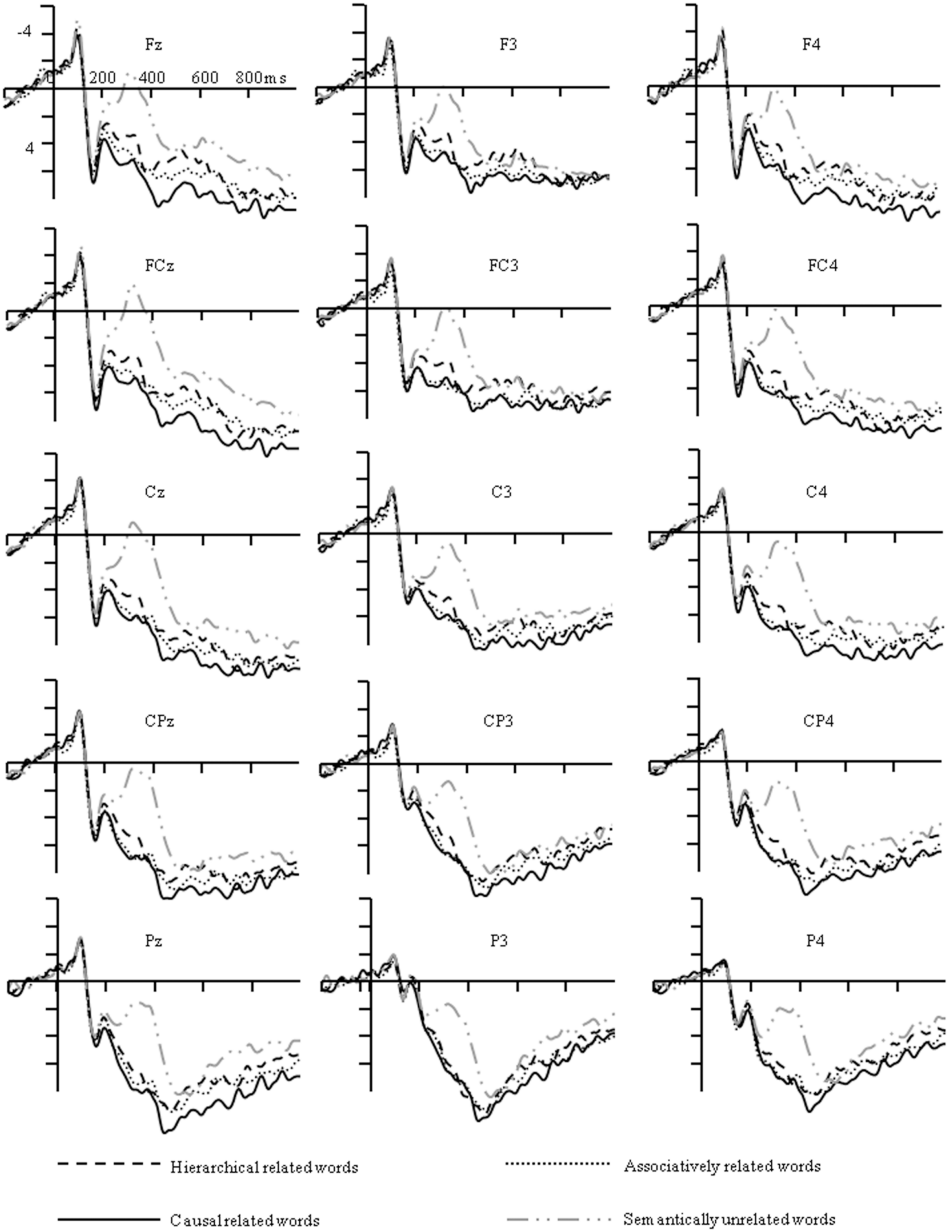
Grand average ERPs at Fz, FCz, Cz, CPz and Pz for different conditions.

As shown in Fig 3, there were significant differences in the amplitudes of the N400 elicited by different types of relations, *F* (3, 42) = 18.75, *ε* = .80, *p* < .001, *η* ^*2*^ = .57. Post hoc pair-wise comparison indicated that the N400 elicited by unrelated words was larger than hierarchically related words (*p* = .005), causally related words (*p* < .001), and associatively related words (*p* < .001). No other difference was found among different types of relations, *ps*>.10.

The main effect of frontality was also significant, *F* (4, 56) = 5.18, *ε* = .34, *p* = 0.03, *η* ^*2*^ = .27. Post hoc pair-wise comparison indicated that the N400 amplitudes at central sites were more negative than central parietal sites (*p* = .06). No other differences were found among them, *ps*>.18. However, there was no significant difference among left, middle and right sites, *F* (2, 28) = .39, *ε* = .69, *p* = .61, *η* ^*2*^ = .03. Furthermore, the type of relation did not interact with the frontality and laterality factors, *F* (12, 168) = 1.49, *ε* = .25, *p* = .23, *η* ^*2*^ = .10, *F* (6, 84) = 2.47, *ε* = .31, *p* = .11, *η* ^*2*^ = .15, respectively.

### Frontal negativity (500–700 ms)

There were significant differences in the mean amplitudes of the frontal negativity elicited by different types of relations, *F* (3, 42) = 3.81, *ε* = .79, *p* = 0.03, *η* ^*2*^ = .21. Post hoc pair-wise comparison indicated that the late ERP component elicited by causally related words was more positive than for hierarchically related words (*p* = .01). Although the mean difference between causally related and unrelated words (1.97μV) was larger than the mean difference between causally related and hierarchically related words (1.49μV), only marginally significant difference was found between causally related and unrelated words (*p* = .08). No other differences were found between them.

The main effect of frontality was also significant, *F* (4, 56) = 3.77, *ε* = .49, *p* = 0.04, *η* ^*2*^ = .21. Although the absolute data of mean amplitudes at frontal (6.14μV) and frontal- central sites (6.69μV) were more negative than central (7.47μV), central-parietal (7.77μV) and parietal sites (7.18μV), post hoc pair-wise comparison indicated that no significant differences were found among them, *ps*>.17. However, the main effect of laterality was not significant, *F* (2, 28) = 1.07, *ε* = .92, *p* = .35, *η* ^*2*^ = .07. Furthermore, the type of relation did not interact with the frontality and laterality factors, *F* (12, 168) = .63, *ε* = .27, *p* = .61, *η* ^*2*^ = .04, *F* (6, 84) = 1.42, *ε* = .37, *p* = .26, *η* ^*2*^ = .09, respectively.

**Fig 4:**
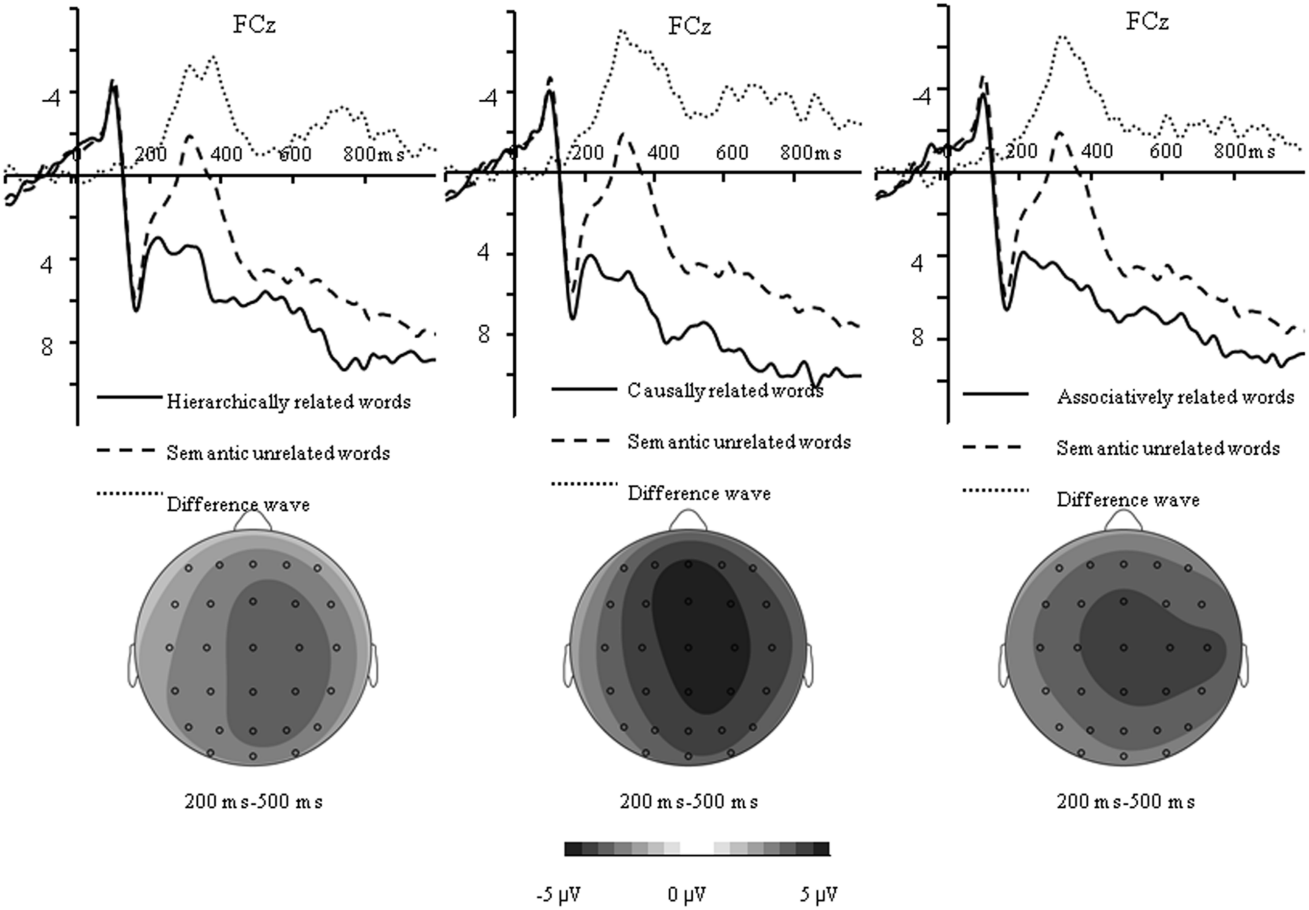
Difference wave and topographical maps for N400 amplitude measured by subtracting the ERP to hierarchically related words (left) from the ERP to unrelated words, and by doing the same with the ERP to causally related words (middle), and associatively related words (right).

**Fig 5:**
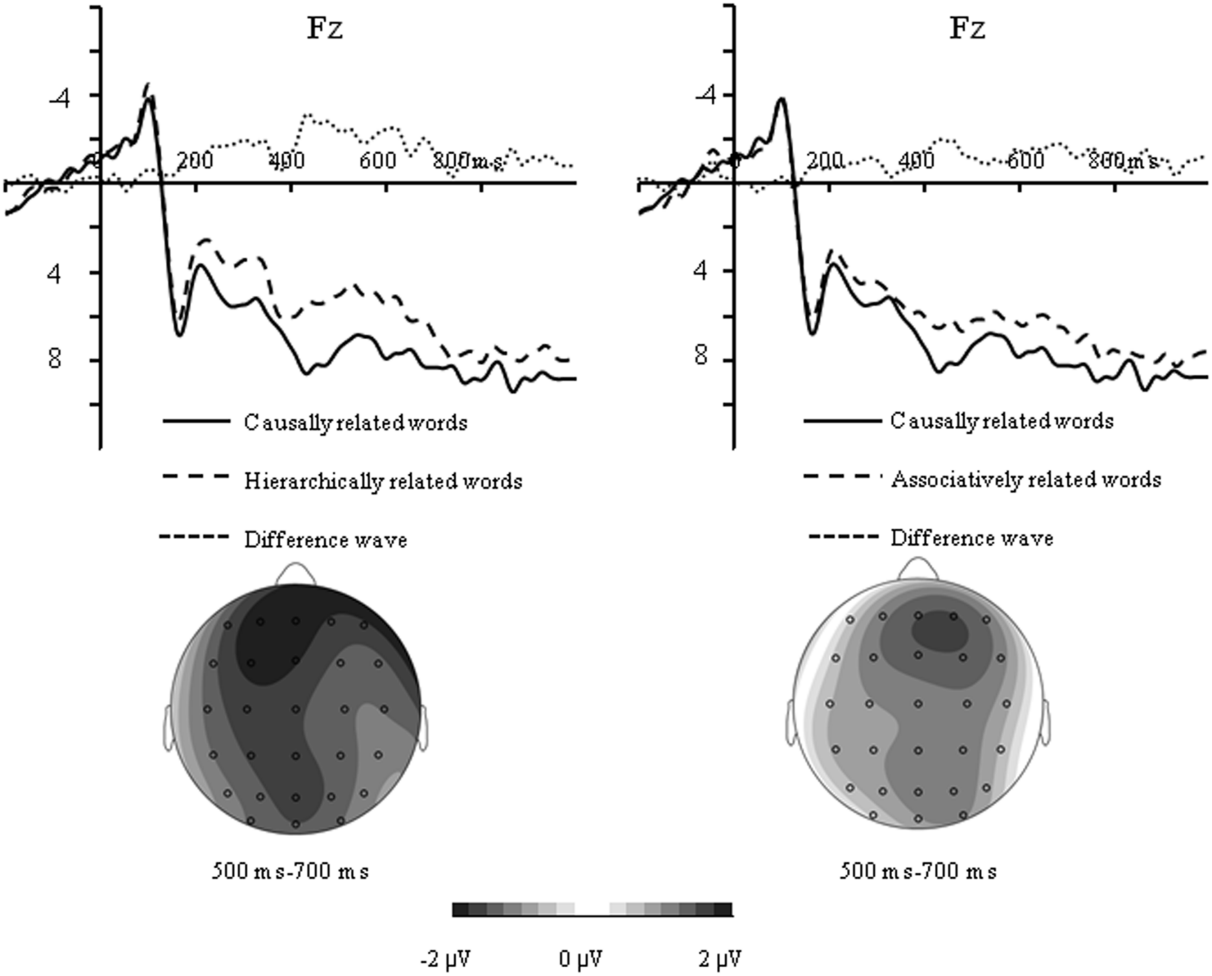
Difference wave and topographical maps for the frontal negativity measured by subtracting the ERP to causally related words from the ERP to hierarchically related words (left), and from the ERP to associatively related words (right).

As shown in Figs 4 and 5, the N400 amplitude elicited by unrelated words was larger than all related words, which was maximally different at central-parietal sites for all related conditions, whereas the difference on the frontal negativity elicited by causally related condition was mainly found at frontal sites.

## Discussion

The main purpose of this study was to investigate the processing of causal relations and hierarchical relations using a general relationship judgment task. Behavioral data found that the accuracy for hierarchically related words was significant higher than for either causally or associatively related words. However, significantly RT difference was only found for hierarchically related words and unrelated words, but not for causal relations and unrelated words. These results were consistent with our previous study [11], and further suggested that the processing of causal relations, another type of thematic relations [13], might involve additional strategic decisions. This finding was in accordance with the spreading activation model [32], which is initiated by a set of source nodes (e.g. concepts) with weights and then iteratively spreading this activation out to other nodes linked to the source nodes. In present study, the first word would have caused a pre-activation of its neighboring nodes storing features or class members. If the second word was related to the activated features or class members, the access to it would be facilitated and the relationship judgment would be accelerated. This process worked in the hierarchical relations, whereas this pre-activation would have been reduced in the causal relations condition because the causally related word pairs are stored in distant nodes.

This interpretation is further supported by the ERP data. Similar N400 effects were evoked by all related word pairs, which were smaller (more positive) than the unrelated pairs. Furthermore, the frontal negativity elicited by causal relations was smaller than that elicited by the hierarchical relations, but not by associative relations. Taken together, our results indicate that the representation of causal relations was dissociated from hierarchical relations, and the processing of causal relations might involve additional processes, such as role binding and prediction processes [17,18].

Previous studies have found that N400 amplitude is sensitive to the strength of semantic relations [23,24]. For example, Kuperberg et al. found that the N400 was smallest for directly causal related sentences, greater for indirectly causal related sentences, and biggest for non-causal related sentences, while keeping semantic association constant across conditions [27]. Overall, the N400 is a reliable index of semantic processing difficulty [24,28]. When the word pairs are unrelated, as in our unrelated condition, the result is an increased N400. After controlling the associative strengths and co-occurrence probabilities of the stimuli, the equal N400 effects were found among causally related, hierarchically related and associatively related words, whereas the N400 elicited by unrelated words was larger than all related words, which is consistent with how the N400 should behave [11].

The main finding of this study was that the frontal negativity elicited by causally related words was smaller than hierarchically related words, but not for general associative related words. The frontal negativity could be seen as a lingering N400-like (or frontal N400) effect [11,28,33], which was directly related to feelings of familiarity [34], or associated with conceptual priming [28]. Our finding is consistent with previous studies, which suggested that the processing of causal relation, maybe another types of thematic relations, involve additional processes, such as role binding and prediction processes [17,18]. For example, Satpute et al. (2005) found greater activation in left dorsolateral refrontal cortex known to role binding for causal judgments relative to associative judgments, and Fenker et al. (2010) found network overlaps with the mesolimbic and mesocortical dopaminergic network known to code prediction errors were more activated by causally related words than by noncausally associatively related words [17]. With regard to the former, maybe additional working memory was required to form a representation in which specific events are bound to the roles of ‘cause’ and ‘effect’ when evaluating the causal relations [18,35]. Furthermore, as the representation of causal relations is asymmetric [14,15], assessing the veracity of causal relations not only depend on sampling associative priming like general associative relations, but also need bind the relevant events into the ‘cause’ and ‘effect’ roles. With regard to the latter, it is probable that the prediction processing is involved in the semantic system, and participants can predict the second word from the first word for causally related word pairs [17,36]. That is, participants can predict the second word from the first word for causally related words, whereas it is more difficult to make such prediction for hierarchically and associatively related words. We are more inclined to interpret the obtained effects to be the result of role binding, because there was no significant difference on the strength of statistical contingency between causally related words and hierarchically related words.

This view is in line with our previous view about the processing of hierarchical relations and productive relations [11]. When different types of semantic relations are investigated using semantic priming in a lexical decision task, an additional memory processing might be involved in the processing of productive relations (a specific type of thematic relations, e.g., bee—honey, bees produce honey), because the apprehension of thematic relations is uncontrollable [12]. As mentioned earlier, like productive relation, the causal relation was also regarded as an example of thematic relations [13]. Thus, when the conceptual relations are investigated in relationship judgment task, the causal relations in such word pairs help bind the words together. The additional involvement of role binding would make it more familiar by conceptual priming, thus, eliciting a smaller frontal negativity.

As mentioned above, our main goal was to examine the ‘implicit’ processing of causal relations and hierarchical relations using a general relationship judgment task, in which participants were required to evaluate whether the two words were related in any way. This was done to tone down the influence of causal asymmetry on our results, because no RT advantage was found for the processing of causal relations in general relationship judgment task [14]. A related line of research should focus on the ‘explicit’ processing of causal related words when participants were required to decide whether two words were causally related, rather than general associated. In fact, we have compared the processing of causally related words with general associatively related words by requiring participants to assess whether the relationship between subsequently presented words matched the initial cue (causal or associative). The results found that the N400 effect elicited by causal relations was smaller than associative relations in causal cue condition, whereas no significant difference was found in the associative cue condition [37]. Once more, the late ERP effect (500–700 ms) was found between types of task cues, but not between causally related and associatively related word pairs. Taken together, these results suggested that the processing of causal relations requires active consideration of the role binding.

According to this study, the representations of causal related words and hierarchical related words are dissociative. However, some issues need to be further studied. For example, the behavioral disadvantages in the processing of causal relations may be associated with a smaller involvement of the frontal negativity. However, no significant correlation was found between the mean amplitude of frontal negativity and the mean RTs for hierarchically related, causally related words, or for unrelated words, *ps*>.22. Furthermore, several studies have explored the asymmetrical representations of causal relations, hierarchical relations, and unidirectional associative strength [14–16]. However, the present study only explored the causal relations in “cause-effect” order or hierarchical relations in “basic-subordinate level” order, and the neural basis of this issue is still unknown. As the spatial resolution of ERP is rather low, additional studies should combine the ERP recordings with fMRI recordings to explore this issue.

## Summary and Conclusions

The present findings yielded new insights into the representation of causal relations in semantic memory, and further suggested the processing of causal relations dissociates from hierarchical relations, but not for general associative relations. Specifically, in addition to a larger N400 effect elicited by unrelated words, the main finding of this study was that the frontal negativities elicited by causally related words were smaller than hierarchical related words, but not for associative related words. These results indicated that the processing of causal relations and hierarchical relations was dissociative, and recruited different degrees of cognitive resources, especially for role binding.

## Supporting Information

S1 Table Normed Causally Related and Hierarchically Related Word Pairs Used in the Experiments. (PDF)

S2 Table Normed Associative Word Pairs and Unrelated Word Pairs Used in the Experiments. (PDF)
