# Supplementary material for: The Processing of Causal and Hierarchical Relations in Semantic Memory as Revealed by N400 and Frontal Negativity
Source: PLoS One. 2015 Jul 6;10(7):e0132679. doi: 10.1371/journal.pone.0132679 (PMC4493067; doi:10.1371/journal.pone.0132679)
Supplement: S1 Table — (PDF) [file pone.0132679.s001.pdf]

S1 Table. Normed Causally Related and Hierarchically Related Word Pairs Used in the Experiments.

Causally related word pairs		English translations		Hierarchically related word pairs		English translations	
硫酸	腐蚀	acid	corrosion	衣物	夹克	clothes	jacket
酒精	事故	alcohol	accident	工具	铁锤	tools	hammer
攻击	防御	attack	defense	花卉	牡丹	flower	peony
细菌	感染	bacteria	infection	珠宝	项链	jewelry	necklace
巨响	耳聋	bang	deafness	食物	土豆	food	potatoes
敲打	伤痕	beat	bruise	住宅	公寓	tenement	flats
背叛	怀疑	betrayal	distrust	疾病	麻疹	disease	measles
犯罪	逮捕	crime	arrest	躯体	手臂	body	arm
疾病	注射	disease	injection	调料	酱油	condiment	sauce
节食	饥饿	diet	hunger	行星	火星	planet	mars
干旱	饥荒	drought	famine	宠物	小狗	pet	puppy
药品	止痛	drug	relief	鞋袜	球鞋	footwear	sneakers
间谍	叛国	espionage	treason	酒类	啤酒	liquor	beer
肥料	生长	fertilizer	growth	鱼类	金鱼	fish	goldfish
黑帮	暴乱	gang	riot	蘑菇	香菇	fungus	mushrooms
燃气	爆炸	gases	explosion	鸟类	鹦鹉	birds	parrot
基因	秃顶	genes	baldness	仪器	天平	instrument	scales
黄金	财富	gold	wealth	体操	双杠	gymnastics	parallel Bars

月球	潮汐	moon	tide	药材	人参	medicinal material	ginseng
损伤	疤痕	lesion	scar	昆虫	甲虫	insect	beetle
闪电	火灾	lightning	fire	油脂	菜油	grease	rape oil
高温	汗水	humidity	sweat	电器	冰箱	domestic appliance	refrigerator
疾病	治疗	illness	treatment	容器	水桶	container	bucket
磁铁	吸引	magnet	attraction	水果	苹果	fruit	apple
笑话	逗乐	joke	amusement	家具	沙发	furniture	sofa
鬼片	噩梦	movie	nightmare	厨具	汤勺	kitchen ware	spoon
变异	癌症	mutation	cancer	蔬菜	白菜	vegetable	cabbage
定购	发货	order	delivery	车辆	货车	vehicle	truck
恐慌	逃走	panic	escape	武器	手枪	weapon	gun
悲伤	哭泣	sadness	crying	乐器	钢琴	musical instrument	piano
食盐	口渴	salt	thirst	文具	钢笔	stationery	pen
刮伤	献血	scratch	blood	媒体	广播	media	broadcast
震惊	尖叫	shock	scream	树木	樟树	trees	camphor tree
扭伤	红肿	sprain	swell	饮料	橙汁	drink	orange juice
压力	疲劳	stress	fatigue	器官	心脏	organ	heart
糖果	龋齿	sweets	cavity	茶叶	绿茶	tea	green tea
锻炼	健康	training	fitness	影视	电影	film and TV	film
垃圾	臭味	trash	stink	家禽	母鸡	poultry	hen

重伤	昏迷	trauma	coma	田径	长跑	track and field	long-distance race
病毒	瘟疫	virus	epidemic	球类	足球	footwear	football
