# Supplementary material for: The Processing of Causal and Hierarchical Relations in Semantic Memory as Revealed by N400 and Frontal Negativity
Source: PLoS One. 2015 Jul 6;10(7):e0132679. doi: 10.1371/journal.pone.0132679 (PMC4493067; doi:10.1371/journal.pone.0132679)
Supplement: S2 Table — (PDF) [file pone.0132679.s002.pdf]

S2 Table. Normed Associative Word Pairs and Unrelated Word Pairs Used in the Experiments.

Associatively Related word pairs		English translations		Unrelated words		English translations	
营销	公司	agency	firm	盆地	学院	basin	academy
篮球	球队	basketball	teams	篮子	风筝	basket	kite
卧室	家具	bedroom	furniture	画笔	骰子	brush	dices
汽车	飞机	car	plane	气泡	丝绒	bubble	velvet
爪子	猎狗	claw	dogs	厨师	害怕	chef	fear
匕首	打架	dagger	fight	小丑	地图	clown	map
礼仪	尊重	decency	respect	玻璃	急流	glass	rush
海港	水兵	harbour	seaman	草地	拳头	grass	fist
电梯	地板	elevator	floor	体操	蔬菜	gymnastic	vegetables
电邮	附件	email	attachment	海港	车库	harbor	garage
羡慕	钦佩	envy	admire	保险	冰糕	insurance	ice cream
家庭	兄妹	family	sibling	标点	女王	point	queen
珊瑚	暗礁	corals	Reef	海报	汉堡	posters	hamburger
玻璃	窗户	glass	window	背叛	蟑螂	revolting	roach
典礼	礼服	graduation	gown	饼干	耳朵	cookie	nose
图表	数字	graph	numbers	沙发	面团	couch	dough
乡镇	城市	town	city	跳舞	液体	dancer	liquid
拳头	指节	fist	knuckles	小鹿	铅笔	deer	pencil

领导	团队	leadership	team	茶水	图表	tea	graph
报纸	八卦	newspaper	gossip	雨水	拳师	water	boxer
公园	风景	garden	landscape	形状	铝材	shape	aluminum
仁慈	同情	kindness	sympathy	制服	插头	smock	plug
跳棋	象棋	checkers	chess	树木	女仆	tree	maid
病人	医生	patient	doctor	钻石	齿轮	diamond	gear
强盗	小偷	robber	thief	唱片	地面	disk	ground
文件	信封	paper	envelope	钻孔	客人	drill	guest
馅饼	汉堡	patty	hamburger	老鹰	儿童	eagle	child
功率	电压	power	voltage	鸡蛋	说谎	eggs	liar
寺院	和尚	temple	monk	引擎	手套	engine	glove
戒指	翡翠	ring	emerald	风景	数学	landscape	maths
安全	部队	security	force	榜样	曲线	lead	curve
学期	课程	session	course	柠檬	足球	lemon	soccer
形状	弧形	shape	curve	豹子	河流	leopard	river
小虾	海洋	shrimp	ocean	公里	围裙	mile	apron
小说	章节	story	passage	奇迹	生姜	miracle	ginger
情侣	女友	lover	girlfriend	老鼠	光线	mouse	light
制服	军官	uniforms	officers	页码	礼服	page	tuxedo
车辆	摩托	vehicle	bicycle	父母	天气	parents	weather
血管	静脉	vessel	vein	屋顶	海员	roof	seaman
剃刀	刀片	razor	blade	野蛮	机场	savage	airport
